# Circulation of Human Coronaviruses in SARS-CoV-2 and Influenza-negative patients with respiratory infections during Ghana's 2021 COVID-19 pandemic

**DOI:** 10.4314/gmj.v60i1.4

**Published:** 2026-03

**Authors:** Mildred A Adusei-Poku, Emmanuella A Apuri, Loretta Kwasah, Linda Boatemaa, Yaw Awuku-Larbi, Joseph A Quarcoo, Nana A Asante Ntim, Ivy A Asante

**Affiliations:** 1 Department of Medical Microbiology, University of Ghana Medical School, College of Health Sciences, University of Ghana, Legon, Accra, Ghana; 2 Virology Department, Noguchi Memorial Institute for Medical Research, College of Health Sciences, University of Ghana, Legon, Accra, Ghana

**Keywords:** Human coronaviruses (HCoVs), respiratory tract infections, COVID-19, pandemic, Ghana

## Abstract

**Objective:**

To determine the prevalence and distribution of human coronaviruses among persons presenting with respiratory symptoms during the COVID-19 pandemic in Ghana.

**Design:**

A retrospective cross-sectional study

**Setting:**

Outpatients and inpatients with respiratory tract infections during the COVID-19 pandemic in Ghana

**Participants:**

A total of 350 samples were collected from persons presenting with respiratory symptoms between July and December 2021. These samples were previously tested and confirmed negative for both SARS-CoV-2 and influenza viruses.

**Intervention:**

None

**Main outcome:**

It was expected that human coronaviruses (HCoVs) 229E, NL63, OC43, and HKU1 would be detected among SARS-CoV-2 and influenza-negative patients presenting with respiratory symptoms.

**Results:**

Of the 350 samples analysed, HCoVs were detected in 123 (35%). Among these, 83% (102/123) were from outpatients, with 17% (21/123) from inpatients. The distribution of specific HCoVs was as follows: 22% (78/350) tested positive for NL63, 5% (18/350) for OC43, 4% (14/350) for HKU1, and 4% (13/350) for 229E. The highest detection rate was observed in the 15–24-year age group, accounting for 46% (56/123) of positive cases, followed by the 25–44-year group at 29% (36/123). The lowest detection rate was noted in infants under 1 year old. Additionally, eight cases (2%) showed co-detections of different HCoVs, with NL63 co-infections being the most common.

**Conclusion:**

HCoVs 229E, NL63, OC43, and HKU1 were prevalent in our study population. This study underscores the importance of differential diagnoses for respiratory viruses with pandemic potential.

**Funding:**

This study was funded by a fellowship from the West African Genetic Medicine Centre (WAGMC/2021/0009).

## Introduction

Acute respiratory infections (ARIs) are common conditions affecting the respiratory tract, ranging from mild upper respiratory tract (URT) symptoms to severe lower respiratory tract (LRT) diseases, such as pneumonia.^1^,^2^ A wide range of viral pathogens are implicated in ARIs, including coronaviruses, picornaviruses, orthomyxoviruses, paramyxoviruses, human metapneumovirus, adenoviruses, and human bocaviruses.^1^–^3^ Coronaviruses (CoVs) belong to the family Coronaviridae, subfamily Coronavirinae, and are classified into four genera. Among humans, alpha-CoVs include human coronavirus 229E (HCoV-229E) and human coronavirus Netherland 63 (HCoV-NL63), beta-CoVs [(HCoV-organ culture 43 (OC43) and HCoV-Hongkong university 1 (HCoV-HKU1)].^4^–^6^ These human coronaviruses (HCoVs) are known to cause ARIs associated with non-specific influenza symptoms and have been identified in various sub-regions, including Africa. 7 They are transmitted by contact with respiratory droplets from an infected person's cough or sneeze, aerosol, contaminated fomite, and asymptomatic carriers.^8^,^9^

Previous studies in Ghana have linked HCoVs and upper respiratory tract infections (URTIs) among individuals with severe acute respiratory infections (SARI).^10^,^11^

During the COVID-19 pandemic, individuals frequently presented with febrile illnesses and respiratory symptoms but tested negative for SARS-CoV-2 and influenza viruses. Given that HCoVs and influenza viruses can cause symptoms similar to those of SARS-CoV-2, it is plausible that symptomatic samples testing negative for SARS-CoV-2 and influenza viruses could have been positive for other HCoVs. The study aimed to determine the prevalence and distribution of HCoVs, specifically HCoVs 229E, NL63, OC43, and HKU1, at the height of the COVID-19 pandemic in Ghana.

## Methods

### Ethical clearance

This retrospective cross-sectional study was approved by the Ethics and Protocol Review Committee (EPRC) of the College of Health Science of the University of Ghana (Protocol identification number CHS-Et/M.9 - P5.14/2022-2023).

### Sample selection

This study utilised previously characterised respiratory samples from patients presenting with symptoms of COVID-19 that were archived at the Noguchi Memorial Institute for Medical Research (NMIMR) in Ghana. During the study period (July–December 2021), a total of 1,738 samples were received and tested for SARS-CoV-2 and influenza viruses. Of these, 325 samples were excluded because they were either positive for SARS-CoV-2 or influenza viruses or had incomplete demographic information.

From the remaining pool of 1,413 SARS-CoV-2 and influenza-negative samples, 350 were randomly selected across all age groups for inclusion in this sub-study, regardless of underlying health conditions. The random selection process was conducted to ensure representative sampling and minimise bias.

The minimum required sample size was calculated to be 167, based on an expected prevalence of 12.4% and a precision of 5%. However, 350 samples were included to increase statistical power and reduce potential confounding or sampling error.

### Viral RNA isolation

The NX-48S Viral NA assay, an automated extraction system, was used to isolate and purify viral RNA from selected samples, following the manufacturer's instructions. Briefly, 200 µL of each specimen was added to the lysis buffer compartment on a plate. This was subsequently placed into the Nextrator (NX-48S) for RNA isolation. Cellular components were initially lysed, exposing RNA, which subsequently bound to magnetic beads. Bound RNA was then purified. RNA was then released into an elution buffer. The plate was removed from the machine and placed on a magnetic stand for 10 minutes, after which 60µl of purified RNA was transferred to the corresponding labelled microcentrifuge tubes.

### Detection of human coronaviruses nucleocapsid gene using Real-Time Reverse Transcription Polymerase Chain Reaction

We used Eurofins Genomics Europe Synthesis primers and probes targeting the nucleocapsid (N) gene, a conserved coronavirus gene. Following the manufacturer's instructions, we performed real-time reverse transcriptase polymerase chain reaction using the ABI 7500 RTPCR system (Life Technologies, Grand Island, NY, USA). Negative and positive controls were included in each run. Cycle threshold (Ct) values were interpreted as either positive (Ct values between 15 and 39) or negative (Ct value of 40 or undetected target) per the PCR protocol.

### Data analyses

Data were analysed using Stata version 16 and summarised as categorical variables, with frequencies and percentages. A graph was plotted to show the distribution of PCR-positive results from the selected sites over time. The positivity rates of HCoVs (OC43, NL63, HKU1, and 229E) and demographic characteristics (sex, age group, patient types, region and symptoms) were compared using Pearson's chi-squared test. The null hypothesis was that there was no significant difference in HCoV positivity rates across categories of each characteristic, and p-values less than 0.05 were considered statistically significant.

## Results

Between July and December 2021, 350 archived patient samples were selected and tested for the presence of human coronaviruses. The study had an equal gender distribution, with participants aged 1 month to 98 years, with a median age of 24 years (interquartile range: 16-34 years). Out of the 350 samples, 123 (35%) tested positive for at least one of the HCoVs. Specifically, 78 (22%) were positive for NL63, 18 (5%) for OC43, 14 (4%) for HKU1, and 13 (4%) for 229E. Most HCoV-positive cases were outpatient, with the predominantly affected age group being 15-24, thus 46% (56/123). Significant gender and age group variations were observed for OC43 and HKU1 and OC43 and 229E, respectively. The most common symptoms exhibited by infected patients include fever, cough, sore throat and headache. Detailed results are presented in Table 1.

**Table 1:** Socio-demographics and Positivity of HCoVs in Ghana July-December, 2021

Characteristics	Total (N)	OC43		P-value	229E		P-value	HKU1		P-value	NL63		P-value
		n	n(%)		n	n%		n	% (n/N)		n	% (n/N)	
Total	350	18	5%		13	4%		14	4%		78	22%	
**Age group, years**													
**Less than 1**	4	1	25%	0.007	0	0%	0.043	0	0%	0.709	0	0%	0.896
**1-4**	25	2	8%		1	4%		1	4%		6	24%	
**5-14**	34	3	9%		0	0%		0	0%		7	21%	
**15-24**	114	10	9%		10	9%		7	6%		29	25%	
**25-44**	133	2	2%		2	2%		5	4%		27	20%	
**45-59**	21	0	0%		0	0%		0	0%		5	24%	
**60+**	19	0	0%		0	0%		1	5%		4	21%	
**Sex**													
**Male**	175	14	8%	0.016	8	5%	0.396	11	6%	0.029	43	25%	0.304
**Female**	175	4	2%		5	3%		3	2%		35	20%	
**Patients Type**													
**Outpatients**	281	16	6%	0.346	13	5%	<0.001	12	4%	0.602	61	22%	0.600
**Inpatients**	69	2	3%		0	0%		2	3%		17	25%	
**Symptoms**													
**Fever**													
**Present**	177	10	6%	0.664	4	2%	0.146	6	3%	0.556	43	24%	0.361
**Not present**	173	8	5%		9	5%		8	5%		35	20%	
**Cough**													
**Present**	214	11	5%	0.998	4	2%	0.022	7	3%	0.383	53	25%	0.162
**Not present**	136	7	5%		9	7%		7	5%		25	18%	
**Sore Throat**													
**Present**	104	4	4%	0.475	4	4%	0.932	5	5%	0.616	20	19%	0.372
**Not present**	246	14	6%		9	4%		9	4%		58	24%	
**Headache**													
**Present**	40	1	3%	0.421	0	0%	0.187	0	0%	0.17	4	10%	0.372
**Not present**	310	17	5%		13	4%		14	5%		74	24%	

### Regional distribution of HCoVs Positivity in Ghana, July-December 2021

**Figure 1:**
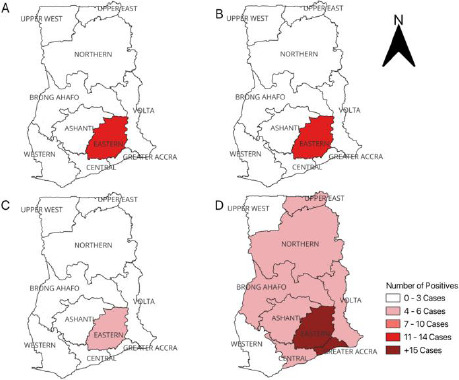
Map of Ghana showing the distribution of HCoVs detected from 350 archived respiratory samples. A. OC43 B. 299E C. HKU1 D. NL63

Of the 123 positive samples, the Eastern region recorded the highest number of HCoV infections, with 57 cases, followed by the Greater Accra region with 27. The Ashanti region reported 9 cases, while the Volta, Upper East, Western, and Northern regions each reported 5 to 6 cases. The Brong Ahafo and Central regions both reported 4 cases, while the Upper West region recorded the lowest, with just 1 case. Among the four HCoV strains investigated, HCoV-NL63 was the most prevalent nationwide, with the majority of infections occurring in the Eastern and Greater Accra regions. The Upper West region recorded the lowest HCoV infections, as illustrated in Figure 1.

### Codetection of multiple HCoVs in Ghana, July-December 2021

Co-infection of multiple HCoV strains was observed in 8 samples involving 7 outpatients and 1 inpatient. This included 4 samples positive for both HKU1 and NL63 (3 outpatients, 1 inpatient), 3 samples positive for OC43 and HKU1 (all outpatients) and 1 sample positive for OC43 and 229E (1 inpatient) as detailed in Table 2.

**Table 2:** Co-detection of multiple HCoVs in Ghana, July-December 2021

Region	Total	Co-detection		

		229E/OC43	HKU1/NL63	NL63/OC43
**Eastern**	6	1	2	3
**Greater**	2	0	2	0
**Accra**				
**Total**	**8**	**1**	**4**	**3**

### Trends in the detection of HCoV positivity per month

HCoVs were detected in both wet (July to October) and dry seasons (November to December). The highest detection rates occurred during the wet season, with 16% of outpatients and 5% of inpatients testing positive. In contrast, during the dry season, detection rates were lower: 11% of outpatients and 1% of inpatients tested positive. The most frequently detected strain, HCOV-NL63, showed the highest detection rates from week 42 (18th-24th October 2021) to week 47 (22nd-28th November 2021), peaking in week 32(9th-15th August 2021). The lowest detection rates for HCOV-NL63 were observed between weeks 27 (5th to 11th July 2021) and 30 (26th July to 1st August 2021).

**Figure 2:**
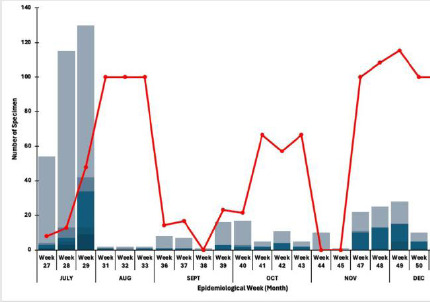
Trends in the detection of HCoVs positivity in SARS-CoV-2 and Influenza negative per month

HCoV-OC43 had its highest detection rate between weeks 42(18th-24th October 2021) and 44(1st-7th November 2021), with a peak in week 31(2nd-8th August 2021). Notably, there were no detections of HCoV-OC43 during weeks 33(16th-22nd August 2021), 34(23rd-29th August 2021), 39(27th September to 3rd October 2021), 41(11th-17th October 2021), 44(1st-7th November 2021), and 47(22nd-28th November 2021). For HCoV-HKU1, highest detection rates were recorded between weeks 43(25th-31st October 2021) and 47(22nd-28th November 2021) peaking in week 30(26th July to 1st August 2021). In contrast, HCoV-229E had its highest detection rate early in the wet season, between weeks 27 (5th to 11th July 2021) and 29 (19th-25th July 2021), without a distinct peak (Figure 2).

## Discussion

Human coronaviruses significantly contribute to ARIs with the emergence of highly pathogenic SARS-CoV-2, drawing renewed attention to endemic strains such as 229E, HKU1, NL63, and OC43.^7^,^12^ This study analysed archived respiratory samples from individuals who tested negative for influenza viruses and SARS-CoV-2 during the COVID-19 pandemic to determine the prevalence and distribution of circulating HCoVs in Ghana during the period.

In this study, HCoVs were detected in 35% of respiratory samples that were negative for both SARS-CoV-2 and influenza viruses. The patients were positive for at least one of the four HCoVs: 229E, HKU1, NL63, and OC43. This finding demonstrates that endemic HCoVs continued to circulate during the height of the COVID-19 pandemic, potentially contributing to respiratory symptoms among individuals who tested negative for the pandemic and seasonal influenza viruses. A similar study conducted in Senegal identified the same four HCoVs during surveillance of influenza-like illnesses, though at a lower prevalence of 4.3%. 7 The higher prevalence observed in our study could reflect differences in study design (our focus on SARS-CoV-2 and influenza-negative cases), time period, population immunity, or seasonality; all of which may have influenced viral circulation patterns.

An earlier study from Ghana reported a lower HCoV prevalence of 12.4%. 10 The higher detection rate in the present study may be due to differences in sampling period, diagnostic sensitivity, or the increased respiratory virus surveillance during the COVID-19 era. Nonetheless, both findings indicate that HCoVs have remained endemic in Ghana over time, reinforcing the need for continued surveillance and the incorporation of HCoV testing into the differential diagnosis of respiratory infections.

Among the HCoVs detected, NL63 was the predominant strain, followed by OC43, HKU1, and 229E. This finding is consistent with reports from South Africa showing frequent detection of HCoV-NL63.^13^ While previous studies have suggested that prior exposure to HCoV-NL63 may provide cross-reactive immunity to other coronaviruses^14^-^16^, our study did not assess immune responses; therefore, this remains a possible explanation requiring further investigation.

HCoVs were detected across all age groups, with individuals aged 15–24 years showing the highest frequency of infections. This trend may be related to greater social contact or mobility in this age group, as suggested by studies elsewhere^16^, but our data do not directly measure behavioural factors; thus, this interpretation should be viewed as a plausible but untested hypothesis. Variations in strain distribution across age groups were also noted, with HCoV-OC43 infections more frequent in infants under 1 year and HCoV-229E more common among young adults. These patterns could reflect differences in immune maturity or prior exposures; however, causality cannot be inferred from this molecular data alone.

Although males and females were equally represented in the study population, males had higher HCoV detection rates, particularly for OC43 and HKU1. This observation aligns with prior studies suggesting potential gender-related differences in susceptibility.^17^–^20^ Possible biological and behavioural factors, such as hormone-related immune modulation or healthcare-seeking behaviours, may contribute, but these mechanisms were not evaluated in this study and therefore remain speculative.

Codetections were identified in 2% of samples, with NL63/HKU1 combinations being the most common. The detection of multiple HCoVs in individual samples indicates that co-infection can occur, consistent with previous studies.^7^, ^10^, ^13^, ^21^, ^23^ Given the high recombination potential of coronaviruses, such co-detections may facilitate viral evolution and the emergence of new variants^24^,^25^, though our study did not include genomic analyses to confirm this.

Regional variation was observed, with the Eastern Region recording the highest number of infections, followed by the Greater Accra Region. Environmental factors such as humidity, temperature, and population density may influence the circulation of HCoV.^31^,^33^ However, differences in testing capacity, healthcare access, and public health interventions across regions could also account for the observed distribution. The low number of detections in the Upper West Region may reflect reduced population density and lower transmission potential.^36^

HCoVs were detected during both wet and dry seasons, with NL63 observed year-round, in line with prior studies showing its ability to circulate across seasons.^24^ The predominance of HCoV-OC43 during the harmattan period may relate to climatic factors such as low humidity and high dust exposure. 10 Nonetheless, without longitudinal data, our study cannot establish definitive seasonal trends.

This study has several limitations. First, its retrospective cross-sectional design limits the ability to infer causality or temporal relationships. Second, while 1,738 samples were initially tested for SARS-CoV-2 and influenza viruses, only 350 SARS-CoV-2- and influenza-negative samples were included in the HCoV testing, introducing potential selection bias and limiting generalizability. Third, the absence of clinical and demographic data (e.g., disease severity, symptoms, comorbidities, or outcomes) prevented assessment of correlations between HCoV detection and clinical illness. Fourth, asymptomatic carriage of HCoVs could not be ruled out; hence, detection does not necessarily indicate causation of respiratory symptoms. Fifth, the study did not include testing for other common respiratory pathogens such as RSV, human metapneumovirus, adenoviruses, and parainfluenza viruses, which may have been co-circulating or responsible for some of the observed illnesses. Sixth, we conducted multiple chi-squared tests, increasing the risk of type 1 error, as some significant values may be due to chance. Therefore, future studies should employ multiple comparisons to enhance the reliability of their results. Finally, the lack of immune, behavioural, and hormonal data precludes direct testing of the mechanisms proposed to explain observed patterns.

## Conclusion

This study demonstrated the continued circulation of HCoVs 229E, NL63, OC43, and HKU1 among patients with respiratory symptoms who were negative for SARS-CoV-2 and influenza viruses during the COVID-19 pandemic in Ghana. The findings highlight the importance of ongoing surveillance of endemic respiratory viruses and inclusion of HCoVs in diagnostic panels, particularly in the context of overlapping respiratory infections with pandemic potential.
